# In vitro–in vivo correlation of drug release profiles from medicated contact lenses using an in vitro eye blink model

**DOI:** 10.1007/s13346-022-01276-6

**Published:** 2022-12-17

**Authors:** Ana F. Pereira-da-Mota, Maria Vivero-Lopez, Piyush Garg, Chau-Minh Phan, Angel Concheiro, Lyndon Jones, Carmen Alvarez-Lorenzo

**Affiliations:** 1 Departamento de Farmacología, Farmacia y Tecnología Farmacéutica, I+D Farma Group (GI-1645), Facultad de Farmacia, Instituto de Materiales (iMATUS) and Health Research Institute of Santiago de Compostela (IDIS), Universidade de Santiago de Compostela, 15782 Santiago de Compostela, Spain; 2 Centre for Ocular Research & Education (CORE), School of Optometry and Vision Science, University of Waterloo, Waterloo, ON Canada; 3 Centre for Eye and Vision Research (CEVR), 17W, Hong Kong Science Park, Hong Kong

**Keywords:** Drug-eluting contact lenses, Eye blink model, In vitro–in vivo correlations, Pravastatin sodium, Resveratrol

## Abstract

There is still a paucity of information on how in vitro release profiles from drug-loaded contact lenses (CLs) recorded in 3D printed eye models correlate with in vivo profiles. This work aims to evaluate the release profiles of two drug-loaded CLs in a 3D in vitro eye blink model and compare the obtained results with the release in a vial and the drug levels in tear fluid previously obtained from an animal in vivo study. In vitro release in the eye model was tested at two different flow rates (5 and 10 µL/min) and a blink speed of 1 blink/10 s. Model CLs were loaded with two different drugs, hydrophilic pravastatin and hydrophobic resveratrol. The release of both drugs was more sustained and lower in the 3D eye model compared to the in vitro release in vials. Interestingly, both drugs presented similar release patterns in the eye model and in vivo, although the total amount of drugs released in the eye model was significantly lower, especially for resveratrol. Strong correlations between percentages of pravastatin released in the eye model and in vivo were found. These findings suggest that the current 3D printed eye blink model could be a useful tool to measure the release of ophthalmic drugs from medicated CLs. Nevertheless, physiological parameters such as the composition of the tear fluid and eyeball surface, tear flow rates, and temperature should be optimized in further studies.

**Graphical abstract:**

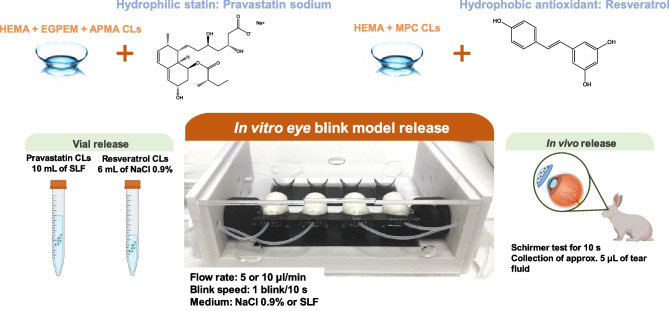

## Introduction

The use of contact lenses (CLs) as platforms for controlled delivery of ophthalmic drugs was envisioned in 1961 by Otto Wichterle and co-workers [1]. After 60 years, the first commercial drug-delivering CL has become available in Japan, Canada, and the USA [2]. Compared to eye drops, CLs may significantly extend drug residence time and increase ocular bioavailability, while unproductive drug absorption is minimized [3].

CLs are one of the most successfully commercialized biomedical devices, with nearly 150 million users worldwide [4]. However, several obstacles oppose their use as platforms for ocular drug delivery [5]. Most polymers used to prepare CLs, e.g., 2-hydroxyethyl methacrylate (HEMA) and 3-(methacryloyloxy)propyl tris(trimethylsiloxy)silane (TRIS), lack affinity for drugs; thus, typically, CLs do not uptake the required dose or release it too rapidly. A wide variety of strategies to overcome this issue is under development [6–9]. Additionally, there are no standardized methods for testing in vitro the drug release profiles from CLs. In vitro methods for mimicking the composition and dynamics of tear fluid, and the frequency and pressure of blinking are still a challenge. Thus, in most reports focused on CLs, the in vitro drug release profiles are recorded in small beakers using a variety of medium composition, volume, stirring, and replacement conditions [10, 11]. As a consequence, most in vitro results are not predictive of in vivo performance [12]. This means that in vivo testing in animal models and human preclinical studies are still needed, even to evaluate early-stage drug-CL combination products, which makes the development very costly in time and resources.

In vitro models that mimic the in vivo scenario and key ocular parameters are highly explored. Microfluidic devices have been designed to regulate the flow and volume where CLs are immersed, but other physiological conditions were not reproduced by these devices, including factors such as corneal and eyelid shape and format, tear film thickness, or blinking [13, 14]. 3D printed in vitro eye models to evaluate the in vitro performance of CLs have recently been undertaken to overcome some challenges faced by using microfluidic devices and more appropriately simulate the effects of tear flow rate, tear volume, air exposure, and eyelid blinking frequency [15, 16]. Not surprisingly, under dynamic conditions of low tear fluid flow, CLs showed slower drug release profiles compared to static release in a beaker, prolonging the release for days or weeks for some specific compounds and drugs such as red food dye [16], polyethylene glycol, hydroxypropyl methylcellulose [17], moxifloxacin [18], and fluconazole [19]. Moreover, in vitro models can provide relevant insights in the development process of drug-loaded CLs and prioritize successful materials that may go forward to in vivo testing in animal preclinical models or human clinical studies.

There is still a paucity of information on how in vitro release profiles recorded in 3D printed eye models correlate with in vivo profiles. Comparison of the behavior of the same drug-loaded CLs in both the in vitro model and the common rabbit eye model is, therefore, required for the validation of the information gathered in vitro*.* To gain an insight into the in vitro–in vivo correlations, the aim of this work was to analyze the release profiles of drug-loaded CLs recorded in a 3D printed in vitro eye blink model and compare the obtained results with the release in a small beaker and the tear levels previously obtained in vivo. For the sake of robustness, CLs loaded with drugs differing in physicochemical properties were tested, namely, CLs designed to uptake pravastatin (a hydrophilic statin, log *P* =  − 0.23 [20]) and resveratrol (a highly hydrophobic antioxidant, log *P* = 3.09 [21]) were prepared and evaluated [22, 23].

**Fig. 1:**
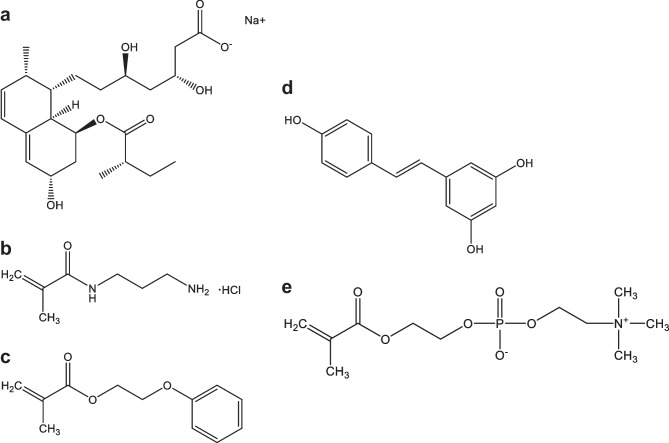
Chemical structures of the drugs and monomers. **a** Pravastatin sodium, **b** N-(3-aminopropyl) methacrylamide hydrochloride (APMA), **c** ethylene glycol phenyl ether methacrylate (EGPEM), **d** *trans*-resveratrol, and **e** 2-methacryloyloxyethyl phosphorylcholine (MPC)

Pravastatin sodium and resveratrol may be useful for the treatment of a wide range of anterior and posterior ocular diseases. Prolonged oral therapy for hypercholesterolemia with statins has been shown to promote corneal healing, prevent cataract formation, reduce glaucoma severity, and reduce the appearance of hard exudates and microaneurysms in patients diagnosed with diabetic macular edema; topical ocular treatment has the advantage of avoiding systemic adverse reactions [24–27]. Resveratrol is an antioxidant agent that aids the management of oxidative-stress-related eye diseases and improves the healing of corneal epithelial cells [28]. In previous studies, both drugs were incorporated in model CLs, and the in vivo performance was evaluated in New Zealand white rabbits [22, 29]. Both drug-loaded CLs provided significantly higher and more prolonged drug levels in the rabbits’ tear fluid compared to eye drops with the same dose, which favored ocular biodistribution in the anterior and posterior structures of the eye, including cornea, sclera, lens, aqueous and vitreous humors, and retina. To carry out the present work, HEMA-based CLs were copolymerized with specific functional monomers that enhance drug affinity. In the case of pravastatin, HEMA was copolymerized with ethylene glycol phenyl ether methacrylate (EGPEM) and N-(3-aminopropyl) methacrylamide hydrochloride (APMA) (Fig. 1). For resveratrol, methacryloyloxyethyl phosphorylcholine (MPC) was added as an antifouling comonomer. The developed CLs, coded as AECLs and MCLs, demonstrated adequate solvent uptake, light transmission, mechanical properties, and ocular safety. In vitro release experiments were carried out with sterile CLs loaded under specific conditions for each drug depending on their physicochemical properties. In vitro release in the 3D eye blink model was tested at two different tears fluid flow rates (5 and 10 µl/min of fluid) and a blink speed of 1 blink/10 s. The amount of drug released from the CLs was collected and quantified by high-performance liquid chromatography (HPLC). To the best of our knowledge, this is the first time that drug-loaded CL release profiles have been evaluated in an in vitro 3D eye model and in vitro–in vivo correlations (IVIVC) are attempted.

## Materials and methods

### Materials

Pravastatin sodium was supplied by Biocon Limited (Bengaluru, Karnataka, India). Resveratrol was from ChemCruz, Santa Cruz Biotechnology Inc. (Dallas, TX, USA). Di-sodium hydrogen phosphate anhydrous (NaH_2_PO_4_) and 2-hydroxyethyl methacrylate (HEMA) were from Merck (Darmstadt, Germany). N-(3-aminopropyl) methacrylamide hydrochloride (APMA) was from PolySciences Inc. (Warrington, PA, USA). Ethylene glycol dimethacrylate (EGDMA), ethylene glycol phenyl ether methacrylate (EGPEM), 2,2′-azobis(isobutyronitrile) (AIBN), 2-methacryloyloxyethyl phosphorylcholine (MPC), polyvinyl alcohol (PVA, 89–98 kDa, 99% hydrolyzed), and dimethyl sulfoxide (DMSO) were from Sigma-Aldrich (Steinheim, Germany). Sodium chloride (NaCl) was from Labkem (Barcelona, Spain), and sodium hydroxide (NaOH) was from VWR Chemicals (Leuven, Belgium). The 3D printing UV-sensitive resin was from Anycubic Technology Co. (Shenzhen, Guangdong, China). Methanol 99.9% for LC–MS grade was from Thermo Fisher Scientific (Loughborough, UK). Simulated lachrymal fluid (SLF) was prepared as previously reported [22]. Ultrapure water (resistivity > 18.2 MΩ cm; Milli-Q^®^; Millipore Ibérica, Madrid, Spain) was obtained by reverse osmosis.

### Contact lens preparation

Two different types of HEMA-based CLs were prepared as previously described [22, 29]. Briefly, AECLs for pravastatin were prepared by mixing HEMA (3 mL) with APMA (21.45 mg), EGPEM (112.50 µL), and EGDMA (12.10 µL). The monomer solutions were magnetically stirred (200 rpm at room temperature) for 120 min, and the initiator (AIBN, 14.79 mg) was then added and solubilized by magnetic stirring for a further 30 min.

To prepare MCLs for resveratrol, HEMA (3 mL) was mixed with MPC (337.5 mg) and EGDMA (12.10 µL) under magnetic stirring (150 rpm at room temperature) for 60 min. The mixture was kept under magnetic stirring for 30 min more to ensure the complete dissolution of AIBN (32.85 mg).

Both AECLs and MCLs were synthesized by adding 60 µL of monomer solution into curved polypropylene moulds typically used for daily disposable CL preparation. The moulds were kept at 50 °C for 12 h and then at 70 °C for 24 h to complete thermal polymerization. Then, the moulds were immersed in MilliQ^®^ water to facilitate CL separation. The obtained CLs were washed under magnetic stirring (200 rpm) in 1 L of MilliQ^®^ water and NaCl 0.9% until complete removal of unreacted monomers occurred; the solvent was replaced at least three times per day. The absence of unreacted monomers was verified by UV–Vis spectrophotometry (Agilent 8453, Waldbronn, Germany).

The final dimensions of hydrated CLs (immersed in phosphate buffer, pH 7.4) were approximately 12 mm diameter, 7.8 mm curvature, and 0.1 mm thickness for AECLs and approximately 14 mm diameter, 8.8 mm curvature, and 0.1 mm thickness for MCLs.

### Drug loading

#### Pravastatin sodium

Dried AECLs (average mass 16.91 ± 1.28 mg) were packaged and sealed in polyamide/polyethylene vacuum bags filled with 10 mL of an aqueous pravastatin solution (0.1 mg/mL) for at least 48 h and sterilized by high hydrostatic pressure (HHP, 70 °C and 600 MPa for 10 min) [30]. The CLs were stored in sealed bags at room temperature and protected from light until release experiments were performed. All the experiments were carried out in quadruplicate. The amount of pravastatin loaded was quantified by HPLC, as explained in “Drug quantification methods” section.

#### Resveratrol

Sterile MCLs (average mass 16.8 ± 1.86 mg, sterilized by steam heat at 121 °C, 20 min) were placed in tubes containing 7 mL of a resveratrol solution (0.1 mg/mL in ethanol:water 10:90 v/v) previously filtered (Filter-Lab^®^ polyethersulphone (PES) syringe filter 0.22 μm; Barcelona, Spain). The loading solution was added to the tubes under sterile conditions in a biological safety cabinet. The tubes were maintained protected from light to avoid resveratrol degradation at 37 °C, 180 rpm for 72 h, after which the release tests were performed (*n* = 4). The amount of resveratrol loaded was quantified by HPLC, as described in “Drug quantification methods” section.

### Eye blink model

**Fig. 2:**
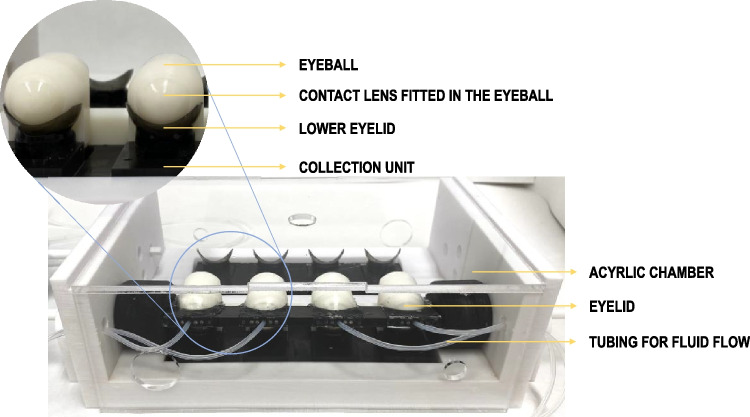
In vitro eye blink model (Ocublink) setup. The eyelid movement spreads the tear solution, which is supplied through the tubing that is attached to the eyelid support, over the eyeball, and the contact lens (fitted on the eyeball, shown in the close-up image). The out-flow solution is collected in the collection unit located below the eyeball

The fabrication and assembly of the 3D eye model were similar to those reported in previous publications by some authors of this paper [15, 16], with minor changes (Fig. 2).

#### Eyeball and collection unit

The eyeball, lower eyelid, and collection unit were fabricated using a combination of 3D printing and moulding techniques, as previously described [31]. The components were printed using a hydrophobic UV-polymerizable resin on an SLA (stereolithography) 3D printer (Photon S; Anycubic, Shenzhen, China) and an FDM (fused deposition modelling) 3D printer (Prusa i3 MK3S + ; Prusa Prague, Czech Republic) to ensure water-sealed parts. All printing parameters were set to the manufacturer’s default settings. The eyeball was composed of two curvatures, 11.25 mm for the larger globe and 8.6 mm for the smaller globe containing the cornea, and 8.6 mm was chosen to match the most common base curve of CLs. Compared to previous models [16], the model used in the current study did not have any coatings for the front corneal surface but instead had a 300-µm groove at the center to allow for a CL to be mounted. In preliminary tests, it was found that the resin materials for the eyeball did not absorb pravastatin and resveratrol. The collection unit of the model was designed to allow the tear film to flow from the eyeball into the wells via gravity.

#### Eyelid

The eyelids were designed to have a curvature of 8.8 mm, which leaves a gap of approximately 200 µm between the eyeball and the eyelid. Once a contact lens was applied on the eyeball, this gap was reduced by the thickness of the lens (75–150 µm). The eyelid was designed to rotate around the smaller globe and flexes over the larger globe.

PVA eyelids were prepared by dissolving PVA (89–98 kDa, 20% w/v) in dimethyl sulfoxide:ultrapure water 80:20 v/v mixtures following a previously described protocol [16]. Briefly, the mixture was gently stirred for 5 min and heated at 120 °C for 2 h. After heating, the mixture was stirred again to ensure proper mixing of PVA. The obtained viscous solution was cast in 3D printed moulds (allowing for the preparation of four eyelids in the same mould) and then frozen at − 30 °C for 12 h. The resulting gels were thawed at room temperature for 1 h, removed from the moulds, and immersed in ultrapure water for 3 days, replacing the medium daily to remove the dimethyl sulfoxide used to prepare the PVA solution. After the washing process, the eyelids were immersed in ultrapure water to maintain the hydration of the eyelid until being used in the release experiments.

#### Flow rate and blinking

A commercial syringe pump (PHD ULTRA; Harvard Apparatus, Holliston, MA) was used to simulate the dynamic tear flow in the eye blink model. Simulated lachrymal fluid (SLF) or NaCl 0.9% was delivered through a hole at the top of the eyelid and spread over the eyeball surface/CL through blinking. In this study, the blink speed was set to 1 blink/10 s, and the flow rate was adjusted to 5 and 10 µL/min to ensure enough volume for sample collection at the predetermined time points. The blink velocity used in this model was 50 rpm or 45 mm/s for both closing and opening speeds; the average physiological speeds for closing and opening of the eyelid have been reported to be approximately 134 ± 4 and 26 ± 2 mm/s, respectively [32].

#### Temperature and humidity

The entire system was covered with an acrylic chamber to maintain stable humidity and temperature (20 ± 1.5 °C) during the experiment. Humidity levels were maintained close to approximately 80% using a humidifier and controlled through a hygrometer.

#### Release sampling

At predetermined time points (5, 15, 30 min, and every hour until 10 h), the out-flow solution was pipetted from the collection unit, stored in 300 or 600 µL Eppendorf^®^ tubes, and frozen at − 30 °C until HPLC analysis. The amount of resveratrol and pravastatin released from the CLs was quantified by HPLC previous dilution of the samples in ethanol:water 50:50 v/v and SLF, respectively. All the experiments were carried out in quadruplicate.

#### Drug extraction from the eyelid and CLs

After 10 h of experimentation, each PVA eyelid was removed from the system, cut into small pieces, and immersed in 1 ml of SLF or 3 ml of ethanol:water 50:50 v/v for AECLs and MCLs. The eyelids were maintained at 37 °C and 180 rpm for at least 12 h to extract the amount of drug absorbed. The same procedure was applied for the CLs after 10 h on the eye model (without cutting) to determine the remaining amount of drug in the CLs at the end of the test.

### Release in a vial

Sterilized pravastatin-loaded AECLs (*n* = 4) were rinsed with SLF to remove excess drug from the CL surface and immersed in 2 or 10 mL of SLF (pH = 7.4) to evaluate if the release volume could have an impact on the drug release profile. The *in vitro* release experiments were performed at 37 °C, under oscillatory movement (180 rpm), and at predefined time points, 150 µL was removed and replaced by the same volume of fresh medium. The amount of pravastatin released from the CLs was quantified by HPLC, as described in “Drug quantification methods” section.

Resveratrol-loaded MCLs (*n* = 4) were rinsed with NaCl 0.9% and placed into 15 mL Falcon^®^ tubes filled with 6 mL of NaCl 0.9%. At each predetermined timepoint, aliquots of 200 μL were removed and replaced with the same volume of fresh medium. After 8 h of release, 6 mL of fresh NaCl 0.9% was added, increasing the total volume of release medium to 12 mL to avoid medium saturation. The amount of resveratrol released from the CLs was quantified by HPLC, as described in “Drug quantification methods” section.

### Drug quantification methods

The amount of pravastatin and resveratrol loaded and released from the CLs was quantified by HPLC using previously developed methods [22, 23]. In vitro release studies in vials were quantified by JASCO HPLC (AS-4140 autosampler, PU-4180 pump, LC-NetII/ADC interface box, CO-4060 column oven, MD-4010 photodiode array detector; JASCO, Tokyo, Japan) operated with the ChromNAV software v.2. In vitro release experiments using the eye blink model were quantified by Waters HPLC (Autosampler Waters 2690, Photodiode Detector 2996; Milford, MA, USA), operated with the Empower2 software.

In the case of pravastatin, the mobile phase consisted of methanol:0.02 M sodium phosphate (NaH_2_PO_4_) buffer (50:50 v/v, pH adjusted to 7.0 with NaOH) at 1.00 mL/min and 25 °C. For pravastatin analysis, both HPLC equipments were fitted with a Waters Symmetry C18 column (5 µm, 3.9 × 150 mm). The injection volume was 80 µL, and the total run time of each sample was 10 min. Pravastatin was quantified at 238 nm (retention time 5.15 min). The HPLC method was validated using pravastatin solutions in simulated lachrymal fluid between 1 and 40 µg/mL.

For resveratrol, the analysis was carried out under isocratic elution using a mobile phase of methanol:water 50:50 v/v at a flow rate of 1 mL/min, 35 °C, and with 8 min of run time. The injection volume was 80 μL, and the UV detector was set at 305 nm. The retention time was 4.6 min. Both HPLC equipment were fitted with a Waters Symmetry C18 column (5 μm, 4.6 × 250 mm). Validation of the method was performed using a calibration curve of resveratrol in ethanol:water 50:50 v/v in the 0.05–6 μg/mL range.

### Statistical analysis

Statistical analysis was performed using Statgraphics Centurion 18 v. 18.1.13 (Statgraphics Technologies, Inc., Warrenton, VA, USA). One-way analysis of variance (ANOVA) followed by multiple range test was carried out. The descriptive data were presented as mean ± standard deviation. In all cases, statistical significance was considered significant for a value of *p* < 0.05.

## Results and discussion

### Three-dimensional eye blink model

In this work, a 3D printed eye blink model was used to mimic some physiological ocular parameters, and in vivo comparisons were carried out. To mimic the tear fluid flow in the eye, fluid flow rates of 5 and 10 µL/min were chosen to ensure there was enough volume for sample collection at every predetermined time point. Preliminary trials carried out with flow rates closer to those reported for physiological tear flow values in humans (1.4–4.3 μL/min, [33]) or in rabbits (0.47–0.66 μL/min [34]) demonstrated that these rates were insufficient to maintain a reliable, constant flow rate of fluid on the eye model required for subsequent sample collections. It should be noted that the experiments with the eye blink model were carried out at room temperature as the system still lacks internal heating.

#### Pravastatin

The amount of pravastatin loaded by the AECLs was approximately 3.50 ± 0.84 mg/g of dried hydrogel. Pravastatin stability against HHP sterilization was previously verified, and the chosen conditions did not trigger degradation compared to non-sterilized pravastatin CLs, which showed that this method is compatible with the sterilization of pravastatin-loaded CLs [30].

**Fig. 3:**
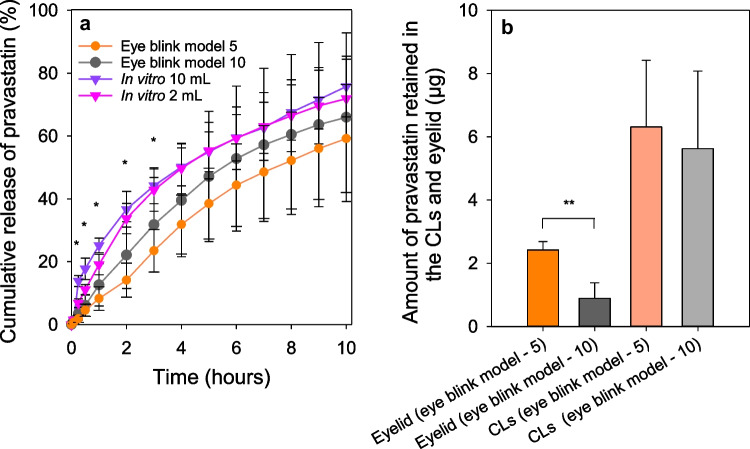
**a** Pravastatin release profiles from AECLs experimentally recorded in a vial filled with 2 or 10 mL of SLF over 10 h and using the eye blink model (flow rate of 5 and 10 µL/min) and **b** amount of pravastatin retained in the PVA eyelid and CLs after 10 h on the eye blink model (*n* = 4, mean values and standard deviations). * Statistically significant differences in the amount of pravastatin released in vitro in 2 and 10 mL and in the eye blink model (flow rate of 5 and 10 µL/min); ** statistically different between the amount of pravastatin retained in the model eyelid with the flow rate of 5 and 10 µL/min, *p* < 0.05

Pravastatin release was investigated in vitro by recording in parallel the amount of pravastatin released when the AECLs were placed in the eye blink model and in test tubes (Fig. 3a). In the in vitro eye blink model, the pravastatin release profiles were more sustained, and the percentage of drug released after 10 h of the experiment was lower compared to in vitro vial release. Statistically significant differences were detected between the percentage of drug released in a vial filled with 2 and 10 mL and the eye blink model (flow rate of 5 and 10 µL/min) in the first 3 h of the experiment (ANOVA, *p* < 0.007). The influence of the flow rate in the eye blink model on the drug released was tested with two different flow rates (5 and 10 µL/min), and a slight decrease in the percentage of pravastatin released was observed with the flow rate of 5 µL/min compared to a flow rate of 10 µL/min, but no statistical differences were detected (ANOVA, *p* > 0.05).

After 10 h of experimentation on the eye blink model, the CLs and eyelids were immersed in SLF in order to measure the amount of pravastatin retained in the materials (Fig. 3b). A significantly higher amount of pravastatin was detected in the PVA eyelid at 5 µL/min compared to the flow rate of 10 µL/min (ANOVA, *p* < 0.001). However, no significant differences were detected in the amount of drug that remained in the CLs for the different flow rates (ANOVA, *p* = 0.72).

For the in vitro vial release, two different volumes were evaluated, 10 and 2 mL of SLF. No statistically significant differences were detected between pravastatin release profiles in 2 and 10 mL of SLF (ANOVA, *p* > 0.05). This minor effect of the volume on the percentage of pravastatin released may be explained by the free-water solubility of pravastatin (40 mg/mL, [35]); therefore, the volume decrease did not induce a false plateau or delayed the release.

The differences between the percentages of pravastatin released in the vial and in the eye blink model could be attributed to several reasons such as the release volume and the absorption of the drug by the eye model materials, among others. Firstly, in the in vitro vial system, the CLs were immediately immersed into vials containing a volume 33 times higher than the volume delivered in the eye model (300 and 600 µL/h). This increase in fluid volume could influence the concentration gradient between the inside of the CL and the release medium, promoting a faster release in the first hours of the release. Secondly, in theory, the CLs in the eye blink model were exposed to a total fluid of 3.0 and 6.0 mL of SLF after 10 h. However, the amounts of fluid collected were 2.23 ± 0.21 mL and 5.47 ± 0.65 mL (for a flow rate of 5 and 10 µL/min, respectively), corresponding to a fluid loss of 25 and 9%. This nonspecific fluid loss due to evaporation, absorption by the eyelid, or dead volume could also contribute to a decrease in the fluid that reached the CL and consequently a decrease in the volume available for drug release. Thirdly, a portion of pravastatin was absorbed by the PVA-eyelid over 10 h of experiment, 2.42 ± 0.26 µg at 5 µL/min and 0.88 ± 0.49 µg at 10 µL/min. As a result, the drug release was slower in the eye blink model, especially in the first few hours.

#### Resveratrol

For resveratrol release experiments, a two-step sterilization protocol was implemented as resveratrol may degrade at high temperature [36, 37]. The MCLs were sterilized by steam heat (121 °C, 20 min) in empty Falcon^®^ tubes, and then the tubes were filled with a previous filtered resveratrol solution (0.1 mg/mL in ethanol:water 10:90 v/v**)**. MCLs loaded, on average, approximately 13.20 ± 0.90 mg of resveratrol per g of dried hydrogel after being immersed in the drug solution for 72 h.

**Fig. 4:**
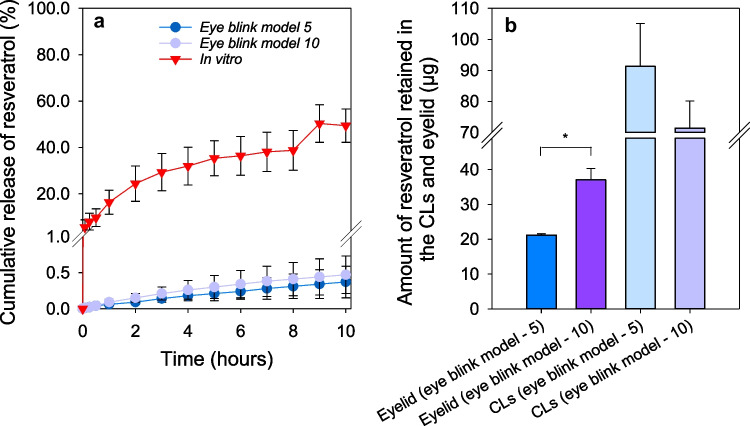
**a** Resveratrol release profiles from MCLs experimentally recorded in a vial filled with 6 mL of NaCl 0.9% over 10 h and on the eye blink model (flow rate of 5 and 10 µL/min) and **b** amount of resveratrol retained in the PVA eyelid and CLs after 10 h on the eye blink model (*n* = 4, mean values and standard deviations). * Statistically different between the amount of resveratrol retained in the model eyelid with the flow rate of 5 and 10 µL/min, *p* < 0.05

In the in vitro vial conditions, the MCLs released 54.43 ± 7.29% resveratrol in the first 10 h (Fig. 4a). As to what happened with pravastatin, the amount of resveratrol released in the eye blink model was significantly lower: 0.37 ± 0.22% at 5 µL/min and 0.47 ± 0.26% at 10 µL/min (ANOVA *p* < 0.001) than the amount released in the vial. The difference between the vial results and eye blink model might be related to the diffusion resistance associated with the hydrophobic nature of resveratrol [38] since resveratrol solubility in NaCl 0.9% was quantified to be approximately 27.4 μg/mL [23]. In the case of the vial tests, the volume of the release medium was increased up to 12 mL to avoid medium saturation and false plateaus.

The amount of resveratrol absorbed by the PVA eyelid was about tenfold higher compared to pravastatin, showing a higher affinity between resveratrol and the PVA eyelid (Fig. 4b). This higher affinity could also contribute to the decrease in resveratrol detected in the fluid collected from the eye blink model. The higher fluid flow rate (10 µL/min) induced a higher amount of resveratrol absorbed by the eyelid (ANOVA, *p* < 0.001). As a consequence of the slow release from the MCLs, the amount of resveratrol remaining in the CLs after 10 h of the release tests was approximately 40% of the drug loaded (91.29 ± 13.85 µg for 5 µL/min and 71.37 ± 8.77 µg for 10 µL/min). No statistical differences were detected between both flow rates (ANOVA, *p* = 0.10).

### In vivo release eye blink model comparisons

The release profiles from the eye blink model for both pravastatin and resveratrol (concentration versus time) obtained were also compared to the in vivo rabbit data already reported for these same CLs [22, 29]. Briefly, six healthy male New Zealand white rabbits were selected for the in vivo release studies wearing drug-loaded CLs. Drug-loaded CLs were removed from the loading solutions, rinsed with sterile saline solution for CLs, and carefully placed on the rabbits’ right eye below the nictitating membrane and without local anesthesia. Samples of the tear fluid were collected using Schirmer test strips before and after CL wearing (*t* = 5, 15, 30 min, and every hour until 8 or 10 h). The drug concentration in the tear fluid was quantified by immersing the Schirmer strips in SLF or ethanol:water (50:50 v/v), and the resulting solutions were quantified by HPLC [22, 29].

#### Pravastatin

**Fig. 5:**
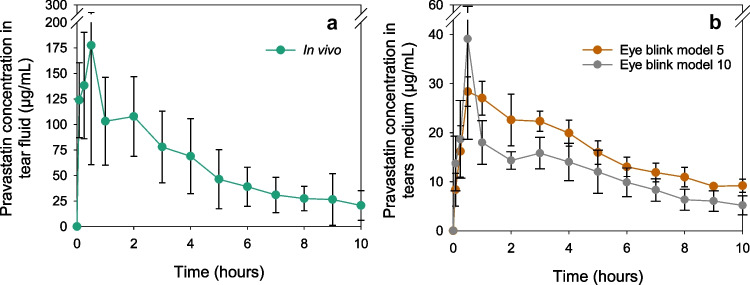
**a** In vivo tear fluid levels of pravastatin were recorded during wear of pravastatin-loaded AECLs for 8 and 10 h (*n* = 6 for 8 h and *n* = 3 for 9 and 10 h), data taken from Pereira-da-Mota et al. [22]. **b** Normalized released pravastatin concentration in SLF from pravastatin-loaded AECLs over 10 h on the eye model (flow rate of 5 and 10 µL/min) (*n* = 4, mean values and standard deviations)

Pravastatin release profiles from the AECLs during the in vivo experiment and in the eye blink model for both flow rates are compared in Fig. 5. The maximum concentration for both experiments was obtained after 30 min of CL application. Pravastatin maximum levels were 177.5 ± 116.8 μg/mL for in vivo and 28.39 ± 3.00 μg/mL and 39.13 ± 20.48 μg/mL for the eye blink model with a flow rate of 5 μL/min and 10 μL/min, respectively. The peak of maximum concentration was followed by a smooth decrease in drug concentration in the tear fluid and in the fluid collected from the eye blink model. No burst release was observed.

Despite the similar release patterns, the amount of pravastatin released from the CLs in the eye blink model was about fivefold lower than that recorded in the in vivo studies (ANOVA, *p* < 0.05). This finding might be related to several factors: (i) the drug absorption into the PVA eyelid in the model, (ii) the composition of the release medium, (iii) the temperature, and (iv) the complexity of the eyeball piece. Firstly, the PVA eyelid absorbed approximately 2.42 ± 0.26 µg pravastatin when tested under 5 µL/min and 0.89 ± 0.49 µg for 10 µL/min. Secondly, the physiological tear fluid contains proteins, lipids, and mucin that can promote drug release from the CLs [39]. In previous studies, the influence of BSA and lysozyme on pravastatin release rate from CLs was evaluated, and an increase in the amount of pravastatin released was observed with the incorporation of both proteins in 2 mL of SLF [22]. The effect of proteins, lipids, and other components of the tear fluid on the release kinetics from CLs is an important aspect to take into account in further studies. Thirdly, in this work, a stable room temperature of 20 ± 1.5 °C was maintained during the release experiments in the eye model, but an increase in temperature from 20 to 34 °C was previously shown to enhance 20% of the fractional mass released from pHEMA hydrogels after 48 h in vitro in vials [40, 41]. This phenomenon could be related to higher kinetic energy of the drug molecules when the temperature increases, leading to faster diffusional transport [42]. The effect of temperature on pravastatin solubility could also contribute to a higher amount of drug released from the CLs in vivo [43]. Fourthly, another limitation that could compromise the release from CLs in the eye blink model pertains to the composition of the eyeball piece. In the present study, the eyeball piece was printed with a hydrophobic UV-polymerizable resin, which does not represent corneal surface properties. Ninety percent of the cornea consists of the stroma, which contains a high percentage of water and consists of collagen fibril lamellae oriented parallel to each other, which is more comparable to a hydrophilic hydrogel. In in vivo conditions, corneal osmosis adds water to the post-lens tear film (the tear film between the back surface of the CL and the corneal epithelium), diluting the concentration of the drug in the post-lens tear film, which could increase the drug release from the drug-loaded CL.

Comparing the release profiles from the different flow rates in the eye blink model, a slightly faster release was observed for the flow rate of 10 µL/min, achieving a higher maximum concentration at 30 min, followed by a decrease in the concentration of drug present in the fluid collected (ANOVA, *p* > 0.05).

**Fig. 6:**
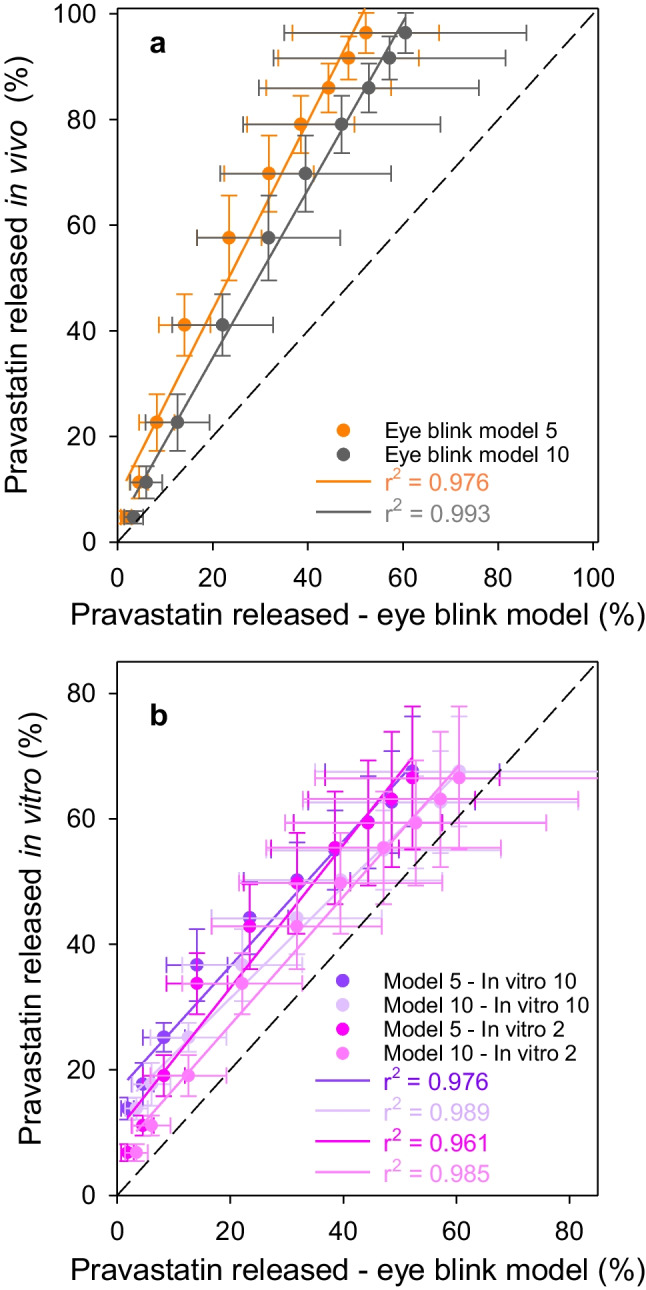
Levy plots for in vivo (**a**) or in vitro (**b**) vs. eye blink model percentage of pravastatin released. The eye blink model experiments were carried out with 5 and 10 µL/min of flow rate (eye blink model 5/model 5 and eye blink model 10/model 10, respectively). The in vitro tests in a vial were carried out in 2 and 10 mL of SLF

Correlations for in vivo-in vitro in the eye blink model or in vitro–in vitro (in a vial versus eye blink model) were investigated using Levy plots (Fig. 6). Release tests in the eye blink model with a flow rate of 10 µL/min led to a correlation coefficient (*r*^2^) closer to 1 (0.993) compared to the Levy plot obtained for 5 µL/min (Fig. 6a). The intercepts at the origin were + 6.25 and + 1.93; the slopes were 1.83 and 1.62 for the flow rate of 5 and 10 µL/min, respectively. Thus, a stronger correlation was observed with the 10 µL/min flow rate that favored the in vivo-in vitro correlations. Also, a higher correlation coefficient was obtained for the Levy plot comparing the in vitro tests in a vial (2 and 10 mL) and eye blink model with the flow rate of 10 µL/min (Fig. 6b).

#### Resveratrol

**Fig. 7:**
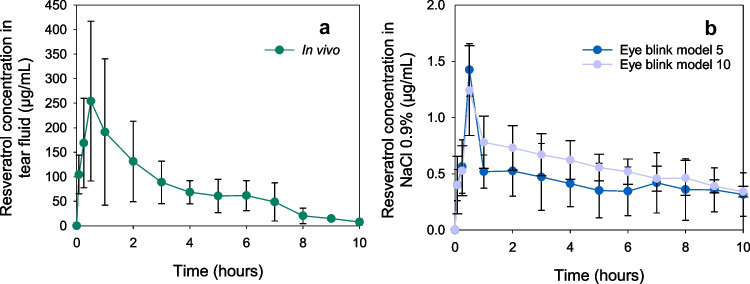
**a** In vivo tear fluid levels of resveratrol were recorded during wear of resveratrol-loaded MCLs for 8 and 10 h (*n* = 6 for 8 h and *n* = 3 for 9 and 10 h), data taken from Vivero-Lopez et al. [29]. **b** Normalized resveratrol released concentrations in NaCl 0.9% from resveratrol-loaded MCLs over 10 h on the eye model (flow rate of 5 and 10 µL/min) (*n* = 4, mean values and standard deviations)

Resveratrol-loaded CLs presented a sustained release in the eye blink model similar to what occurred in vivo (Fig. 7). However, the concentration of resveratrol detected in the fluid collected in the eye blink model was approximately 150-fold lower than in vivo (ANOVA, *p* < 0.001), likely associated with several factors that also conditioned the release of the hydrophilic statin in the eye blink model, plus the low solubility of resveratrol in an aqueous medium, as previously mentioned. No statistical differences were detected between the two different flow rates (ANOVA, *p* > 0.05).

**Fig. 8:**
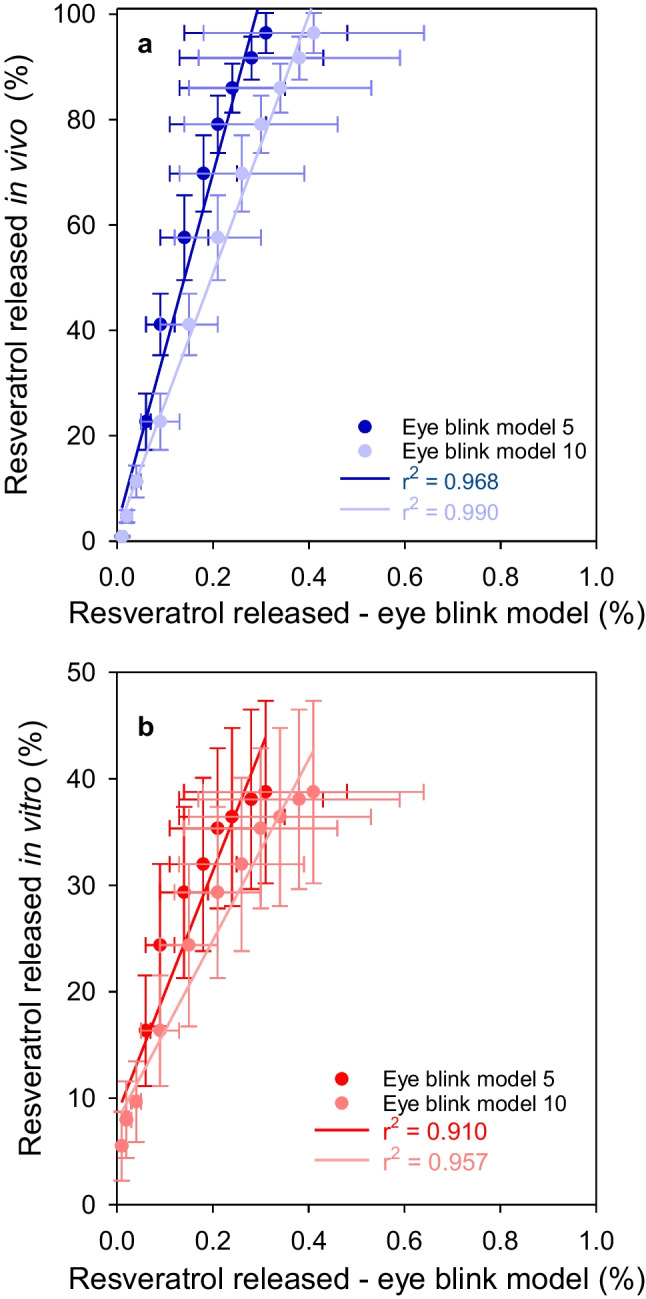
Correlations for in vivo (**a**) or in vitro (**b**) versus eye blink model percentage of resveratrol released. The eye blink model experiments were carried out with 5 and 10 µL/min of flow rate (eye blink model 5 and eye blink model 10, respectively)

For resveratrol experiments, plots of in vivo release versus in vitro release in the eye blink model presented slopes that were remarkably high: 333.96 and 245.16 for the flow rate of 5 and 10 µL/min, respectively (Fig. 8), which supported that the percentage of resveratrol released in vivo was significantly higher than that recorded in the eye blink model. In comparison, no differences were obtained for in vitro vial release and the eye blink model. This finding highlights the difficulties in developing in vitro release models that can predict the in vivo performance of CLs loaded with hydrophobic drugs, which represent about 40% of current pharmaceutical treatments [44].

## Conclusions

In the current study, the usefulness of the developed 3D printed eye blink model for the evaluation of drug release profiles of medicated CLs has been explored in detail. To our knowledge, this was one of the first studies that have attempted to correlate the release of drugs from lenses from an in vivo rabbit study with a blink model. It may serve as an important starting point to understand areas that need to be further developed and investigated to create better simulations. There are numerous factors in the eye that can affect drug release kinetics, and understanding the contribution/effect of each factor individually to drug release kinetics is not obvious. For instance, in this study, increasing the flow rate by 2 times was expected to increase the drug release rate by 2 times. However, this was not the case: increasing the flow by 2 times only resulted in a marginal increase in the amounts of drugs released, which was not obvious. The release profile of both drugs was more sustained and lower in the in vitro eye blink model compared to the in vitro release in vials, especially for the hydrophobic drug resveratrol. Both drug-loaded CLs showed similar release patterns in the eye blink model as in in vivo studies. However, the amount of drug released in the eye blink model was significantly lower compared to previously obtained in vivo data. More linear Levy plots were recorded for pravastatin release in the eye model (flow rate of 10 µL/min) and in vivo data. The information gathered in the present study may serve to gain an insight into relevant physiological parameters that influence the in vivo release from CLs, such as the composition of the tear fluid, tear flow rates, temperature of the system, and composition of the eyeball surface. The obtained results may serve as a guide for further improvements of the 3D printed eye blink model.

## Data Availability

The datasets generated during and/or analyzed during the current study are available from the corresponding author upon reasonable request.
